# Recent and emerging innovations in *Salmonella* detection: a food and environmental perspective

**DOI:** 10.1111/1751-7915.12359

**Published:** 2016-04-04

**Authors:** Rebecca L. Bell, Karen G. Jarvis, Andrea R. Ottesen, Melinda A. McFarland, Eric W. Brown

**Affiliations:** ^1^ Center for Food Safety and Applied NutritionU.S. Food and Drug AdministrationCollege ParkMDUSA; ^2^ Center for Food Safety and Applied NutritionU.S. Food and Drug AdministrationLaurelMDUSA

## Abstract

*Salmonella* is a diverse genus of Gram‐negative bacilli and a major foodborne pathogen responsible for more than a million illnesses annually in the United States alone. Rapid, reliable detection and identification of this pathogen in food and environmental sources is key to safeguarding the food supply. Traditional microbiological culture techniques have been the ‘gold standard’ for State and Federal regulators. Unfortunately, the time to result is too long to effectively monitor foodstuffs, especially those with very short shelf lives. Advances in traditional microbiology and molecular biology over the past 25 years have greatly improved the speed at which this pathogen is detected. Nonetheless, food and environmental samples possess a distinctive set of challenges for these newer, more rapid methodologies. Furthermore, more detailed identification and subtyping strategies still rely heavily on the availability of a pure isolate. However, major shifts in DNA sequencing technologies are meeting this challenge by advancing the detection, identification and subtyping of *Salmonella* towards a culture‐independent diagnostic framework. This review will focus on current approaches and state‐of‐the‐art next‐generation advances in the detection, identification and subtyping of *Salmonella* from food and environmental sources.

## Introduction

Members of the genus *Salmonella*, belonging to the family *Enterobacteriaceae*, are Gram‐negative, non‐spore forming, predominantly motile, facultative anaerobic bacilli. The genus is composed of two species, *enterica* and *bongori*, with six subspecies of *S. enterica* (McQuiston *et al*., 2008). The genus is further subdivided into serotypes based on the presence of specific surface molecules, namely O‐antigen (O‐Ag), present in lipopolysaccharide, and H‐antigen (H‐Ag), typically the major protein of the flagellar complex, flagellin. Collectively, there are over 2500 serotypes of salmonellae, all which are capable of causing disease in humans (Grimont and Weill, 2007).

The majority of salmonellae cause gastroenteritis, however, a few serotypes, such as *S*. Typhi, *S*. Paratyphi A, *S*. Paratyphi B and *S*. Paratyphi C (typhoidal salmonellae), are capable of causing enteric fever, a severe illness characterized by the onset of high fever with abdominal pain and general malaise but not typically with diarrhoea or vomiting. This is in contrast to salmonellosis, a gastroenteritis caused by non‐typhoidal salmonellae, which is characterized by fever, vomiting and severe diarrhoea. In a majority of the cases, salmonellosis is self‐limiting, resolving in about a week. Occasionally, however, the infection becomes systemic, a much more severe disease requiring antibiotic interventions. Yearly in the United States, it is estimated that *Salmonella* is responsible for over a million illnesses, 19 000 hospitalizations and almost 400 deaths (Scallan *et al*., 2011). Studies on the infectious dose of *Salmonella* in humans indicate a wide range for the number of cells required to cause disease. Clinical studies conducted using human volunteers indicate a range of 10^5^ to 10^10^ cells. In contrast, enumeration of food commodities indicate much lower numbers of organisms, as low as 10 cells, were present to cause illness (Blaser and Newman, 1982). Infection typically occurs after the ingestion of contaminated food or water. It is estimated that 95% of *Salmonella* infections are due to the consumption of contaminated foodstuffs (Fatica and Schneider, 2011). These data suggest that salmonellae may be present at very low levels in food and still be able to cause a significant number of infections.

Until the 1990s, most illnesses were due to the consumption of animal products: poultry, poultry products, meat and dairy. Recently, an increasing number of illnesses have been associated with the consumption of raw, fresh, ready‐to‐eat produce, such as tomatoes, melons, sprouts, leafy greens and berries (Painter *et al*., 2013). In most instances, contamination levels in these commodities are quite low and sporadic as demonstrated by several prevalence studies conducted in the United States (Sapers and Doyle, 2014; Bell *et al*., 2015). As such, rapid, reliable and sensitive detection of *Salmonella* in food and environmental sources is essential to safeguard the food supply effectively and subsequently ensure public health.

There has been a thrust in the past 25 years to develop much faster methods to detect, identify and subtype *Salmonella* specifically in food and environmental samples. This review will focus on the current culture‐dependent methods while highlighting some promising innovative culture‐independent methods for the rapid, accurate detection, identification and subtyping of salmonellae in food and environmental samples.

## Culture‐dependent methods

### Currently used method to detect, identify and subtype

**Figure 1:**
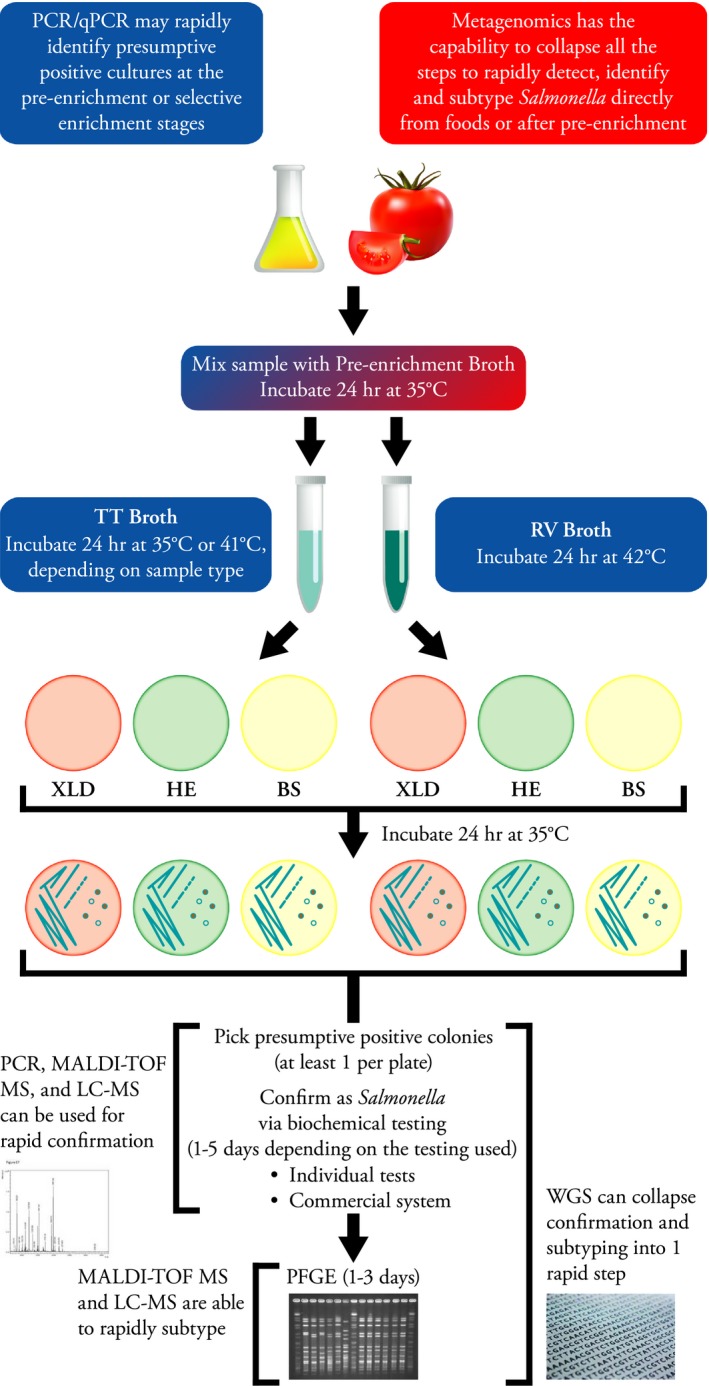
Overview of the U.S. Food and Drug Administration Bacterial Analytical Manual (FDA‐BAM) workflow for the detection, isolation and subtyping of *Salmonella* (Andrews *et al*., 2011). Detection and isolation of *Salmonella* requires 5 days. Subsequent confirmation and subtyping may take up to a week longer. Various, newer molecular methods such as PCR/qPCR, MS, WGS and metagenomics, may shorten the time to result and may be incorporated into the workflow at the indicated steps.

Current testing of food and environmental samples for the presence of *Salmonella* can be divided into three stages: (i) detection of the pathogen; (ii) identification of the isolate as *Salmonella* and its specific serovar designation; and (iii) subtyping of the isolate for association with any clinical cases of salmonellosis. Detection methods rely on traditional bacterial culture procedures that employ the use of serial enrichments with increasing selectivity culminating in the isolation of *Salmonella* on selective‐differential agar plates (Fig. 1) (Andrews *et al*., 2011; USDA FSIS, 2014). The process takes up to 5 days to gather a presumptive positive isolate. Confirmation relies on traditional biochemical testing of sugar and nutrient utilization media, which can take days to complete. Even with newer automated technologies that permit simultaneous testing of multiple analytes at least 24 h is needed for a confirmation of *Salmonella*. DNA finger printing techniques, such as pulsed‐field gel electrophoresis (PFGE), ribotyping and intergenic sequence (IGS) ribotyping have all been used to subtype *Salmonella* isolates. All these techniques are based on a similar idea of examining DNA size differences on an agarose gel. For ribotyping, genomic DNA is digested, separated on an agarose gel and then hybridized to rRNA operons to visualize the banding pattern. After comparison to a database of fingerprints species, serovar and occasionally strain identifications can be made (Bailey *et al*., 2002). More discriminatory power maybe available with IGS ribotyping, where the size differences found within the intergenic spacer regions between 16S and 23S rRNA regions are examined. Here, the regions are amplified by polymerase chain reaction (PCR) before gel electrophoresis is done. The banding patterns allow for differentiation between strains of *Salmonella* within a serovar (Brown, 2001). Neither of these techniques has been widely adopted. Federal and State agencies within the United States and many other countries around the world rely on PFGE to subtype *Salmonella*. For this technique, genomic DNA is digested by the restriction endonuclease *XbaI*. The DNA fragments are separated on an agarose gel subjected to a pulsed electric field. DNA is visualized by ethidium bromide staining and fingerprints are analysed using specific software available in BioNumberics (Applied Maths) (Ribot *et al*., 2006). The power of PFGE is the ability to compare the resultant fingerprint patterns to a large national database housed and maintained by the Centers for Disease Control and Prevention (CDC), aka the PulseNet reference library (Swaminathan *et al*., 2001). The use of PFGE and the implementation of PulseNet have greatly increased the United State's ability of track and trace back illness clusters and outbreaks. Unfortunately, PFGE still requires a pure isolate and a minimum of 3 days to complete (Ribot *et al*., 2006).

While this analytical schema is the ‘gold standard’ of regulatory agencies due to its sensitivity, a detection limit assumed to be 1 cfu per portion of food tested; and its ability to provide a pure culture of Salmonella, many pitfalls remain associated with this approach (Fig. 1). First, as mentioned, the time to result is quite long, taking at least a week to get a confirmed isolate and longer for serotyping and subtyping. For many food commodities, especially fresh produce, this time frame is far too long to effectively test food vehicles before they are consumed or to hold in warehouses while awaiting test results before they spoil. Second, at each step, the amount of media used to find *Salmonella* increases, resulting in numerous plates required for each sample. The process is very labour intensive and necessitates large areas of space, particularly if many samples are being tested. Finally, food samples, especially fresh produce and spices, can be notably difficult for traditional microbiological methods due to the high numbers of indigenous microbiota and the presence of antimicrobials found within the food commodity (Jameson, 1962; Arora and Kaur, 1999; Singer *et al*., 2009; Kim *et al*., 2011; Bell *et al*., 2012; Gorski, 2012; Pettengill *et al*., 2012).

### Advances in detection: PCR and Real‐time PCR

The largest advance towards faster detection of *Salmonella* has been in the realm of molecular biology, where PCR and real‐time, quantitative PCR (qPCR) are predominantly being applied as the methods of choice for the detection stage of this process. Many different protocols targeting different genes or gene regions specific to *Salmonella* have been published (Cohen *et al*., 1996; Guo *et al*., 2000; Malorny *et al*., 2003, 2004; Chen *et al*., 2010; Postollec *et al*., 2011; González‐Escalona *et al*., 2012; Cheng *et al*., 2015). Additionally, many targets have been investigated for the specific and sensitive detection of all salmonellae in food and environmental samples. By far the most popular gene target is *invA*, with some assays multiplexed to improve sensitivity and specificity (Malorny *et al*., 2003; Postollec *et al*., 2011; González‐Escalona *et al*., 2012; Cheng *et al*., 2015). While the obvious advantages of PCR/qPCR assays are the rapid time to result (Table 1), and sensitivity and specificity of detection, there are many disadvantages as well. These include the need for expensive equipment and trained personnel, the use of extensive DNA clean‐up chemistries before addition to the PCR/qPCR reaction, the need to culture the samples to meet the limit of detection threshold, and a lack of distinction between live and dead cells.

**Table 1:** Comparison of traditional methods to molecular methods for *Salmonella* detection, identification and subtyping

Method	Resolving power	Accuracy	Technical competency		Time to result
			Performance	Analysis	
PFGE	Sub‐serotype	100%^d^	Highly trained/Must be certified	Highly trained/Must be certified	1–3 days
Traditional serology	Serotype	Approx. 80%^e^	Highly trained/Must be certified	Highly trained/Must be certified	Up to 3 days
Phage typing^a^	Sub‐serotype	Approx. 80%^f^	Highly trained/Must be certified	Highly trained/Must be certified	1–2 days
PCR/qPCR	Genus to serotype^b^	Varies with protocol and matrix^g^	Moderately trained	Moderately trained	4–6 h
MALDI‐TOF MS	Species	>98% at species level	Easy for clinical workflow	Easy for clinical workflow	<5 min
LC‐MS	Serotype to sub‐serotype level	98% at serotype level^h^	Moderately trained	Highly trained	<1 day
WGS	Strain	100%	Easy to perform	Highly trained	3–4 days^j^
Metagenomics	Genus to strain^c^	Approx. 11%^i^	Easy to perform	Highly trained	3–4 days^j^

a Only used for *S*. Typhi, *S*. Paratyphi A, *S*. Typhimurium and *S*. Enteritidis.

b Depends on matrix and primer sets used, some only detect genus, some will test for specific serotypes.

c Depends on sequencing depth, analysis pipeline and available database to query sequences against.

d If performed within PulseNet certification standards.

e Due to rough, mucoid and non‐motile strains.

f Due to ambiguous lysis reactions.

g Must have a minimum of 10^2^ genomes in the reaction in order to get a positive detection, see text for discussions on other limitations.

h Based on single lab evaluation studies.

i Based on current pipelines and databases in naturally contaminated cilantro (Jarvis *et al*., 2015).

j Depending on analysis time.

Food and environmental samples pose a unique set of challenges for PCR/qPCR reactions. The detection limit of qPCR is approximately 10^2^ cells/reaction. Naturally contaminated food typically will not contain high enough numbers of salmonellae to reach this detection limit in order to attempt direct detection from such food items. This is also true for certain environmental samples such as soils, sediments and waters. As such, there is a need to culture these sample types to enrich for *Salmonella* at levels more easily detected by PCR/qPCR. However, several difficulties may be encountered during enrichment. First, enrichment bias may occur where *Salmonella* cells are outcompeted by the natural microbiota found in the food/environmental sample or the salmonellae are outright inhibited by specialized metabolites, such as antibiotics, produced by these same organisms (Jameson, 1962; Singer *et al*., 2009; Bell *et al*., 2012; Gorski, 2012; Ottesen *et al*., 2013a; Allard *et al*., 2014). Subsequently, the number of *Salmonella* cells may never reach the required threshold for detection. As a result of this bias, the exact time necessary for the enrichment step is largely unknown with suggested ranges of 4–24 h depending on the sample type, the starting concentration of *Salmonella* in the sample and the health of those salmonellae (Myint *et al*., 2006; Josefsen *et al*., 2007; Tatavarthy *et al*., 2009; Cheng *et al*., 2015). Some have even suggested the need to use a secondary, selective enrichment to enhance the chances of detecting *Salmonella* in challenging samples of this nature (Myint *et al*., 2006). Consequently, this greatly lengthens the time to result and reduces the usefulness of PCR/qPCR for faster detection of *Salmonella* in contaminated food and environmental samples. Culturing samples to increase the salmonellae population also increases the background microbiota populations. Unfortunately, the sensitivity of qPCR reactions has been found to decrease in the presence of high concentration of non‐specific DNA suggesting that even higher levels of *Salmonella* must be present in order for detection to occur (Jyoti *et al*., 2011). Compounding this problem, the food or environmental sample may contain PCR/qPCR inhibitors that are not removed during the culture enrichment step. These inhibitors include high concentrations of fats and proteins in meat and dairy as well as polysaccharides and polyphenols in fruits and vegetables (Rossen *et al*., 1992; Wilson, 1997). Humic and fulvic acids may be present in environmental samples, especially soils and sewage, which will prevent PCR/qPCR reactions from occurring (Wilson, 1997). Likewise, the media used for enrichment may contain inhibitors, which also will decrease the sensitivity of the PCR/qPCR reaction (Gorski and Liang, 2010). Protocol adjustments to alleviate the impact of inhibitors inherent to the sample include variation in DNA extraction and clean‐up methods and the addition of facilitators to the PCR/qPCR reaction (Wilson, 1997; Ma and Michailides, 2007; Chua and Bhagwat, 2009). Moreover, internal amplification controls to identify PCR inhibition have become the standard to confirm the efficacy of the sample preparation and clean‐up steps (Malorny *et al*., 2003). Finally, as PCR/qPCR assays are quite sensitive in the detection of DNA molecules, there is a concern over the detection of live versus dead cells because DNA may linger for prolonged periods after the death of the cell. To address this concern, the use of reverse‐transcriptase‐qPCR assays have been developed to detect RNA which will only be produced in living cells (Gonzalez‐Escalona *et al*., 2009). Also the use of propidium monoazide has been proposed as a way to differentiate living from dead cells in a qPCR assay (Li and Chen, 2013).

### Advances in sample preparation to concentrate *Salmonella*

Of all the shortcomings mentioned above, the most problematic is enriching the *Salmonella* population to detectable levels in the limited sample volumes used in PCR/qPCR. There has been a thrust in up‐front processing steps to help selectively separate the *Salmonella* population from the background microorganisms. The most widely used method employs the use of anti‐*Salmonella* antibodies bound to paramagnetic beads. This process, known as immunomagnetic separation (IMS), allows for the specific separation of *Salmonella* from other organisms within the food or environmental sample by first mixing the anti‐*Salmonella* magnetic bead with a portion of the pre‐enrichment culture and then separating them out with the use of a magnet (Cudjoe *et al*., 1994; Shaw *et al*., 1998). This not only decreases the number of background microorganisms but it also concentrates the *Salmonella* to the levels required for detection. This may also allow for shorter pre‐enrichment periods, such as 6 h as opposed to the full 24 h typically used, which would allow detection to occur in 1 day, much earlier than traditional culture methods (Tatavarthy *et al*., 2009). Unfortunately, the sensitivity of IMS is limited by the binding specificity of the antibody to all salmonellae cells – this is critical to prevent false‐negative results as all salmonellae have the ability to cause disease in humans – leaving holes in this method if it is used to screen the food supply (Life Technologies 2012).

Along with antibodies, other biomolecules have been explored as possible means to selectively capture and concentrate *Salmonella* from cultures. Aptamers are single‐stranded oligonucleotides, DNA or RNA that take on unique 3‐D structures based on their primary nucleotide sequence, rendering them capable of binding to specific ligands similar to an antibody interacting with an antigen. Aptamers offer some advantages over antibodies in that they are relatively inexpensive to synthesize, and they provide more batch‐to‐batch consistency (Bruno, 2014). Limited studies have demonstrated their specific use in concentrating *Salmonella* Typhimurium from river water and faecal samples (Joshi *et al*., 2009; Jyoti *et al*., 2011; Singh *et al*., 2012). Bacteriophages have also been explored as a means to capture *Salmonella* cells (Bennett *et al*., 1997; Laube *et al*., 2014). Phages may offer some advantages over antibodies given their inherent specificity for host cells, their ease of production in bacteria versus animals or eukaryotic cell culture and their relative stability in harsh conditions such as pH and temperature extremes (Laube *et al*., 2014).

In a move towards culture‐independent detection others have focused on methods to concentrate all cells within the sample before the pre‐enrichment step. This concentration of the *Salmonella* population allows for direct detection from food and environmental samples. These methods focus mainly on filtering liquids, rinsates, or mechanically disintegrated (i.e. blended or stomached) samples. While this approach has been widely used to test large volumes of water, the testing of food samples was problematic due to clogging of filter membranes by large food particles (Li *et al*., 2013; Vibbert *et al*., 2015). To overcome this problem, endopeptidases have been added to the stomached food samples. These degrade the small, soluble proteins and peptides so that they are unable to clog the filter and pass through with the permeate (Vibbert *et al*., 2015). Additional filtration advances include the use of hollow‐fibre filter membrane cartridges, which have higher surface‐to‐volume ratios than flat‐sheet membranes, as well as cross‐flow, or tangential flow, where the sample flows across the membranes to further aid in the reduction of membrane fouling (Cho *et al*., 2014). This method has recently been awarded the grant prize in the U.S. Food and Drug Administration's first ever Food Safety Challenge competition ( http://www.foodsafetychallenge.com), signifying its potential to greatly enhance the detection of *Salmonella* directly from foods.

### Advances in identification and subtyping: mass spectrometry

Identification of bacteria by mass spectrometry (MS) has been an active research area for decades (Anhalt and Fenselau, 1975). Most MS‐based bacterial identification methods rely on the reproducible patterns generated from measurement of masses of proteins and/or lipids from intact bacterial cells or cell extracts. MS‐based methods are intriguing because they provide direct detection of the presence of expressed bacterial proteins. Tens to hundreds of proteins are measured in a single experiment. The result is a reproducible mass fingerprint with features that are unique to a given genus or species. The significant advantage of MS methods over phenotypic and molecular methods for bacterial identification is that no advanced knowledge of the microorganism is necessary and there is no need for assay selection. The mass spectral fingerprint contains a snap shot of reproducible observable proteins, which makes this an inherently multiplexed screening method.

Matrix‐associated laser desorption ionization–time of flight mass spectrometry (MALDI‐TOF MS) is the most common technique used for bacterial analysis by MS (Claydon *et al*., 1996; Holland *et al*., 1996; Krishnamurthy and Ross, 1996). It is easy to operate, per sample cost of analysis is low, excluding the initial cost of the mass spectrometer, and analysis is as fast as 10 min from colony selection to identification (Table 1). Early work focused on proof of principle of genus and species level differentiation and method standardization (Fenselau and Demirev, 2001; Lay, 2001; Williams *et al*., 2003; Wunschel *et al*., 2005). As multiple laboratories began developing libraries of reference spectra representing species of interest, it became clear that lab‐to‐lab reproducibility was going to require standardization of spectral libraries. In 2008, multiple commercial MALDI‐TOF MS instruments were released that included reference spectra libraries (Martiny *et al*., 2012). Two systems received FDA approval for clinical use in 2013. The research use versions of these systems also include methods for creating libraries customized to the user laboratory. A thorough review of bacterial identification by MALDI‐TOF MS for clinical use can be found in Clark *et al*. (2013).

The critical determinant for accurate identification is the presence of the genus or species of interest in the spectral library. A transferrable spectral library database containing sufficient microbial representation is required for the deployment of an MS‐based assay to multiple laboratories. Most commercially available MALDI‐TOF MS bacterial identification instruments contain databases populated with composite reference spectra from clinically relevant isolates. Consequently, MALDI‐TOF MS is an effective and rapid tool for genus and some species level identifications. Unfortunately, commercially available libraries currently lack the breadth and specificity that is ultimately needed to analyse complex matrices such as food. In addition, commercial identification algorithms struggle or fail to identify mixtures of bacteria. Therefore, a single colony or pure culture is generally required. However, the speed at which even a genus level identification can be made makes MALDI‐TOF MS a potentially important screening tool.

Species level identification of *Salmonella* by MS is relatively straight forward when analysing a pure colony. Comparatively few studies have been published on MS‐based subspecies detection of *Salmonella* (Lynn *et al*., 1999; Leuschner *et al*., 2004; Dieckmann *et al*., 2008; Kuhns *et al*., 2012; Sparbier *et al*., 2012). Ribosomal proteins whose masses are sufficiently different at the genus level dominate MALDI‐TOF MS spectra of bacteria. However, *Salmonella* subspecies and serovar‐level identification continues to be a challenge by MALDI‐TOF MS. MALDI‐TOF MS spectra of complex mixtures suffer from poor spot to spot mass accuracy and peak reproducibility. The current method for MALDI‐TOF MS spectral library creation minimizes these limitations by creating reference spectra that are composites of spectra built from different isolates and different culture conditions while also matching peaks with a wide mass accuracy tolerance. Although this serves to increase reproducibility, it is possible that spectral compositing lowers specificity by masking the ability to detect mass differences created by single amino acid differences. Some specificity can be gained back, however, by adding the appropriate reference spectra to the reference library. Nonetheless, reference libraries populated with too many serovar‐specific reference spectra may challenge commercial matching algorithms because of the high homology across serovars.

Impressive work by Dieckmann and Malorny (2011) has shown that detection of serovar‐specific combinations of proteins is possible by MALDI‐TOF MS. This was accomplished by extending the upper mass range of detection from 20 000 Da up to 40 000 Da. The authors were able to identify several non‐ribosomal serovar‐specific combinations of proteins as markers, but no single marker was serovar‐specific. Combinations of marker masses will be required to achieve such specificity. As an additional caveat, a MALDI‐TOF MS method capable of extending above 20 000 Da is not currently supported by commercial vendors making it difficult to reproduce in other laboratories. As an aside, the authors also identified a large number of the masses that were detected. Such knowledge of the identity of marker proteins being used in reference libraries would go a long way in standardizing across laboratories and instruments.

An alternative to MALDI‐TOF MS is separation and detection of bacterial proteins by high‐performance liquid chromatography mass spectrometry (LC‐MS). LC‐MS of intact bacterial lysates has shown great promise for differentiating closely related species within the *Enterobacteriaceae* family, thermophilic versus non‐thermophilic groups of *Enterobacter sakazaki* and identifying bacterial biomarker proteins for the development of PCR probes (Krishnamurthy *et al*., 1999; Ho and Hsu, 2002; Williams *et al*., 2004, 2005; Mott *et al*., 2010). More recently, LC‐MS of intact lysates has been used for *Salmonella* serovar‐level identification (Callahan *et al*., 2009). This method chromatographically separates the intact bacterial proteins prior to detection by MS. Consequently, many more proteins and, by extension, more serovar‐specific marker proteins are detected. Recent work by McFarland *et al*. (2014) shows that a powerful advantage of this method is that it can be performed on mass spectrometers with improved mass accuracy that are capable of dissociating the proteins so they can be identified. Once the detected proteins are identified they can be used for further analysis such as in silico validation of the markers, extension to unknown isolates and cross‐referencing to genomic‐based assays. Because the proteins are measured intact, this method can recognize changes to detected proteins even if the specific substitution is unknown. This work demonstrates that serovar‐level identification of *Salmonella* is possible by LC‐MS. One disadvantage is that it is slower than MALDI‐TOF MS analysis (Table 1). Nonetheless its robustness at serovar identification and its ability to identify pathogens without an extensive reference library makes this a promising method for further analysis of isolates that are positive for *Salmonella* by MALDI‐TOF MS screening.

### The role of next‐generation sequencing

Next‐generation sequencing (NGS) or whole genome sequencing (WGS) refers to highly automated and parallelized genome sequencers used to sequence the entire genome(s) of bacterial pathogens in a matter of hours. When coupled with analytical bioinformatic pipelines such as the one established at CFSAN‐FDA ( https://github.com/FDA/open.fda.gov), accurate and stable genetic changes can be identified that can distinguish foodborne outbreak strains of *Salmonella* down to the source level including specific farms, food types and geographic regions. Also, the rapid increase in *Salmonella* genome sequences in the NCBI database and other public domains, such as GenomTrakr ( http://www.fda.gov/Food/FoodScienceResearch/WholeGenomeSequencingProgramWGS/default.htm), are changing the way that public health laboratories establish linkages between *Salmonella*, the environment and illness. Moreover, WGS has begun to streamline laboratory testing of salmonellae into a single microbiological workflow, supplanting phenotypic, serologic and other less robust genotypic typing schemes. For example, WGS outputs can now be readily shunted into bioinformatic pipelines that accurately predict antimicrobial susceptibility patterns, determine serotype and provide multiple virulence profiles for a single *Salmonella* strain (Zankari *et al*., 2013; Tyson *et al*., 2015; Zhang *et al*., 2015). In the United States, the National Antimicrobial Resistance Monitoring System has evaluated the use of WGS data to determine the presence of antimicrobial resistance genes. In an initial study, focused on *Escherichia coli*, comparison of WGS data to a database containing over 2500 resistant genes and gene variants was able to predict antimicrobial resistant profiles that highly correlated with phenotypic profiles (Tyson *et al*., 2015). In work conducted in Denmark, WGS data funnelled into the ResFinder web server ( www.genomicepidemiology.org) showed 100% concordance in predicting *S*. Typhimurium resistance profiles when compared with phenotypic data (Zankari *et al*., 2013). The use of WGS data in this way should greatly enhance antimicrobial monitoring systems allowing for better tracking of antimicrobial resistant isolates within the food supply and augmenting epidemiological tracebacks during outbreak situations. Additionally, *Salmonella* serotype may also be predicted from WGS data with the use of the SEQSERO program, recently developed by Deng and colleagues. This simple computer dashboard program quickly extracts both O‐ and H‐antigen types from genomic data quickly predicting serotype with high accuracy when compared with traditional serotyping data (Zhang *et al*., 2015).

Whole genome sequencing is changing the laboratory landscape for foodborne outbreak investigations of *Salmonella* contamination as well. WGS of *Salmonella* strains is supplanting conventional approaches, such as PFGE, which often fall short in ample characterization of a *Salmonella* strain as well as delimiting the scope or tracking the source of contamination potentially associated with such a strain (Allard *et al*., 2012). In certain cases of foodborne contamination, highly clonal strains of *Salmonella* have been known to confound outbreak investigations because conventional subtyping approaches often lack the resolution for differentiating certain closely related isolates (Table 1). As an example, investigations of *S*. Enteritidis outbreaks are taxing because nearly 40% of all egg isolates have the same PFGE pattern, making epidemiological investigations difficult due to a common fingerprint pattern found in a widely distributed food (Allard *et al*., 2013). In response to such challenges, Federal public health and food safety laboratories are exploiting NGS to define complex outbreak scenarios involving *Salmonella*. For example, WGS analysis of *S*. Bareilly isolates responsible for the 2012 tuna scrape outbreak established a clear link between outbreak isolates and a specific manufacturing facility in India (Hoffmann *et al*., 2015). WGS analysis of *S*. Enteritidis isolates from an egg outbreak in the United Kingdom revealed a clear linkage between human, egg and environmental *S*. Enteritidis isolates specific to the outbreak (Inns *et al*., 2015). Additionally, WGS has identified outbreak clusters of *Salmonella* in outbreaks associated with black pepper (*S*. Montivideo), tomato (*S*. Newport), cucumber (*S*. Newport), watermelon (*S*. Newport) and peanut butter (*S*. Tennessee) (Bakker *et al*., 2011; Cao *et al*., 2013; Byrne *et al*., 2014; Angelo *et al*., 2015; Bell *et al*., 2015, M. R. Wilson, unpublished). These genomic studies are providing vital information, retrospectively and in real‐time, for foodborne outbreak investigations. Deployments of WGS have been undertaken using the technology at the U.S. FDA, the CDC, Public Health Canada, Harvard and Cornell Universities, The Sanger‐Wellcome Trust, The Danish Technical University and Danish Food Institutes, numerous public health institutes in Germany, and various industry colleagues engaged in this technology development.

## Culture‐independent approaches

While highly effective for providing important and unique identifiers among individual salmonellae, it is notable that WGS technology, in its most effective form, is requisite on a pure culture for the generation of a complete genome sequence. This caveat makes any current applications for pathogen surveillance or detection directly from complex food matrices or pre‐enriched foods a challenging task. Metagenomic approaches, however, are now beginning to provide a path forward in the use of WGS technology for detection of *Salmonella* in situ in food and environmental backgrounds (Ottesen *et al*., 2013a). Multiple proof‐of‐concept studies for the use of metagenomic methods for pathogen detection have been undertaken in a wide range of fields including the detection of causal agents in human matrices such as plasma, sputum, saliva and faeces (Finkbeiner *et al*., 2008; Rogers *et al*., 2009; Cho and Blaser, 2012; Loman *et al*., 2013; Doughty *et al*., 2014); identifying the causal agents of crop diseases, bee colony collapse; investigation of fermentation processes; and spoilage (Jung *et al*., 2011; Nieminen *et al*., 2012; Delhalle *et al*., 2013; Ma *et al*., 2014; Wanglin and Longhao, 2015). Also the ecologies of poultry, meats, fresh produce and a variety of processed food have all been interrogated using target‐based and/or shotgun metagenomic approaches (Lusk *et al*., 2012; Ottesen *et al*., 2013b; Jackson *et al*., 2015; Jarvis *et al*., 2015).

Metagenomics is defined as the analysis of genomic DNA from a whole community; this distinguishes it from genomics, which is the analysis of genomic DNA from an individual organism or cell (Chen and Pachter, 2005; Gilbert and Dupont, 2011). Definitions vary to include any study that analyses a whole community, for example, from directed studies of 16S rRNA gene diversity within an environment, to isolation and analysis of total DNA from environmental samples without prior cultivation (Chen and Pachter, 2005; Gilbert and Dupont, 2011). A recent publication by Marchesi and Ravel (2015) defines a metagenome as the collection of genomes and genes from the members of a microbiota obtained through shotgun sequencing of DNA extracted from a sample. The microbiota consists of all the microorganisms present in a defined environment as described by amplifying and sequencing specific marker genes, such as 16S rRNA and 18S rRNA (Marchesi and Ravel, 2015). The term metataxomics is proposed for the high‐throughput process used to characterize the entire microbiota by sequencing specific marker genes that can be used to infer taxonomies (Marchesi and Ravel, 2015).

Target‐based bacterial metagenomic studies have focused on the use of 16S rRNA gene sequences. 16S rRNA gene sequencing takes advantage of the ability to use the conserved regions of the 16S rRNA gene to identify bacterial taxa. PCR primers specific to variable regions of the 16S rRNA gene are capable of amplifying all of the 16S rRNA genes present in a sample, followed by high‐throughput sequencing. Samples can be barcoded and multiplexed at various levels depending on the depth of sequencing desired. The 16S rRNA gene sequencing data reveal the bacterial taxonomy of a sample as operational taxonomic units, which can be clustered into similar sequences using software programs such as QIIME, MOTHUR and Resphera Insights ( http://www.respherabio.com/Resphera_Insight_v2.2.pdf) (Schloss *et al*., 2009; Caporaso *et al*., 2010). It is now notable the number of known 16S rRNA gene sequences has surpassed the number of cultured organisms and therefore this analysis reveals the total microbial population in the sample. Further analysis of the taxonomic breakdown of a sample can reveal the proportional abundances of each taxon. For example, taxonomic composition can also be classified according to alpha (within sample) and beta (between sample) diversity algorithms to establish similarities and differences between sample populations (Lozupone and Knight, 2005; Caporaso *et al*., 2010).

In contrast to 16S rRNA studies, shotgun metagenomic studies are valuable for looking deeper into a sample and may allow for genome assembly depending on the abundance of the organism(s) of interest. Additionally, because shotgun metagenomic studies are not target specific, the data reveal information about all domains present in a sample. Uniformly sheared genomic DNA is barcoded and sequenced. Multiplexing considerations are more critical for shotgun metagenomic sequencing as no amplification step is necessary. Subsequently, sequencing depth becomes more important, especially with high diversity samples.

This leads to a discussion of the many details that must be considered before metagenomic approaches become reliable applications for pathogen, specifically *Salmonella*, detection. First, the question of how much sequence data are needed to reliably detect a pathogen, such as *Salmonella*, should be addressed. This is contingent upon species richness and abundance in the food or environmental matrix, the size of all genomes present, the number of salmonellae present in the sample, and the number and length of all the sequences that have been generated. This also depends on the selection of gene targets when using targeted metagenomic approaches. For example, fragments of 16S rRNA genes, such as the popular V4‐V6 variable region and nucleotides 533–1492 at the 3′ end of the molecule, have shown to be useful for inferring taxonomy of bacterial genera and sometimes species; however, there are numerous situations where the taxonomic resolution achievable with these regions is not specific enough to identify *Salmonella* from other genera within *Enterobacteriaceae* (Lane *et al*., 1985; Weisburg *et al*., 1991; Fox *et al*., 1992; DeSantis *et al*., 2006; Caporaso *et al*., 2012). Conversely, recent work has demonstrated that the V1‐V3 region, located at the beginning of the 16S rRNA gene, was able to assign a call of *Salmonella* to the genus level with 99% confidence (Jarvis *et al*., 2015). Next, the question of database quality needs to be examined. Genomic and metagenomic data sets are deposited in national and international repositories. The utility of these data sets is dependent on the quality of the sequence data and whether or not detailed and accurate metadata has been submitted. To identify a sequence as *Salmonella* with high confidence, highly curated genetic signatures and genomes of the target pathogen and other coexisting organisms must be available. Studies that demonstrated the functionality of metagenomic methods to recover pathogens of interest knew the target ahead of time and had the full genome as a reference and/or other diagnostic genetic signatures available (Loman *et al*., 2013; Leonard *et al*., 2015). Finally, the choice of bioinformatic pipelines, which are used to annotate metagenomic data sets, needs to be considered. These pipelines tend to be computationally intense and ultimately rely on well‐curated reference databases and pathogen genomes in order to provide reliable taxonomic descriptions and detections. Each pipeline and reference database must be tailored for each target pathogen and the respective environment from which it is being detected.

Culture‐independent analyses for public health investigations and surveillance are transforming the way samples are screened for genetic content. The ability to sequence all of the genomic material in a sample is changing the way microbial ecologists, clinicians and food scientists analyse and identify organisms of interest.

Recently published studies that used 16S rRNA gene sequencing to detect *Salmonella* in tomato and cilantro, also investigated microbiota shifts during the culture‐based FDA‐BAM method for the detection of *Salmonella* in produce. These studies concluded that the bacterial phyllosphere changed from a predominance of *Proteobacteria*, which includes *Salmonella*, to a predominance of *Firmicutes* after a 24‐h non‐selective pre‐enrichment (Pettengill *et al*., 2012; Ottesen *et al*., 2013a; Jarvis *et al*., 2015). The diversity of the uncultured samples was higher than the cultured samples, and there were inherent beta‐diversity differences between replicate samples for each commodity tested. An interesting finding in the tomato study was that even though RT‐PCR and FDA‐BAM culture methods were negative for *Salmonella*, the bioinformatic analysis of 16S rRNA gene sequences showed putative hits to *Salmonella* (Ottesen *et al*., 2013a). Conversely, in the cilantro study, only one of six *Salmonella* culture‐positive samples contained *Salmonella*‐specific 16S rRNA gene sequences (Jarvis *et al*., 2015). Further characterization of the culture‐positive cilantro metagenomes showed a clear difference in levels of *Salmonella* genomic DNA, suggesting that contamination levels varied among the six culture‐positive cilantro samples tested (Jarvis *et al*., 2015). Both the tomato and the cilantro culture‐independent studies demonstrate the importance of considering the impact of competing microorganisms on our ability to detect and identify pathogens such as *Salmonella* in the complex environments such as food, despite the specific genetic or genomic method being employed.

Metagenomic studies also help to establish microbial baselines for foodstuffs, where a ‘healthy’ microbiome for fresh and processed foods may be defined. These culture‐independent data sets provide valuable starting points to expand current understandings of food ecologies and how they respond to intentional and unintentional perturbations, such as agricultural management practices and contamination events. For example, Ottesen *et al*. (2013b) performed a baseline survey of the tomato microbiome to characterize the tomato phyllosphere. In this study, metataxonomic (16S and 18S rRNA gene sequencing) and metagenomic sequencing were performed on tomato plant parts including roots, bottom leaves, stems, tomatoes, flowers and top leaves. The authors observed a gradient of diversity from low to high going from the top to the bottom of the plant (Ottesen *et al*., 2013b). The tomato and cilantro studies revealed the core microbiomes of these food plants and lay the groundwork for understanding how pathogens such as *Salmonella* survive and thrive in the environment and in foods (Ottesen *et al*., 2013b; Jarvis *et al*., 2015).

Along with pathogen detection, metagenomic studies may be useful to identify co‐culturing microbes. These include native communities that may one day be used as indicator organisms or signature consortia that correlate with risk of pathogen presence, persistence, or virulence. The principle that isolation of a microbe must involve separating it from its surrounding community has formed the basis of culture‐based microbiology. However, a paradigm shift is underway where the recognition that no organism is an island and pathogens exits within complex microbial communities and may rely on population density or other external conditions to express certain virulence traits (Rogers *et al*., 2009).

## Conclusion

In summary, the various methods reviewed here each have utility for *Salmonella* testing in the food safety sector. It is important to recall, however, that not every method will be recommended or even suited for every situation in testing all food varieties for this pathogen. For instance, application to specific food samples will be dictated by method performance. As noted previously, method performance is dependent on several things including matrix driven effects, general sensitivity and specificity of the method, and its technical complexity. Other extrinsic factors include user skill set and technical prowess, cost of the equipment and cost per sample. Ultimately, systematic validation of a method will ascertain its specific utility and application across the food supply.

In considering the current spectrum of rapid molecular methods for detecting *Salmonella*, it is clear that several approaches have emerged including PCR‐based, mass spectrometric and others encompassing those stemming from the current genomics era (see Table 1 for comparison to traditional methods). All of these methods are moving towards greater automation, network integration, with the ultimate goal of a fully automated laboratory test workflow. It is important to recall that outputs from one approach will serve to strengthen other approaches with many of these methods being either directly or tangentially related. For example, the explosion of WGS data is contributing not only to better characterizations of salmonellae from foods but also contributes directly to enhanced PCR‐based screening tools for this diverse genus of foodborne pathogen. Ultimately, it seems that a suite of tools is emerging for the food safety microbiologist, each with its specific strengths and weaknesses but all with the ability to rapidly and accurately detect *Salmonella* with greater specificity and early in its contamination of the human and veterinary food supply.

## Conflict of Interest

The authors declare no conflicts of interest.
